# Dissimilar Appearances Are Deceptive–Common microRNAs and Therapeutic Strategies in Liver Cancer and Melanoma

**DOI:** 10.3390/cells9010114

**Published:** 2020-01-02

**Authors:** Lisa Linck-Paulus, Claus Hellerbrand, Anja K. Bosserhoff, Peter Dietrich

**Affiliations:** 1 Institute of Biochemistry, Emil-Fischer-Zentrum, Friedrich-Alexander-University Erlangen-Nürnberg, 91054 Erlangen, Germany; lisa.linck@fau.de (L.L.-P.); claus.hellerbrand@fau.de (C.H.); 2 Comprehensive Cancer Center (CCC) Erlangen-EMN, 91054 Erlangen, Germany; 3 Department of Medicine 1, University Hospital Erlangen, Friedrich-Alexander-University Erlangen-Nürnberg, 91054 Erlangen, Germany

**Keywords:** miRNA, melanoma, hepatocellular carcinoma, liver cancer, let-7, *miR-622*, *mir-26a*, *miR-221*, *miR-210*

## Abstract

In this review, we summarize the current knowledge on miRNAs as therapeutic targets in two cancer types that were frequently described to be driven by miRNAs—melanoma and hepatocellular carcinoma (HCC). By focusing on common microRNAs and associated pathways in these—at first sight—dissimilar cancer types, we aim at revealing similar molecular mechanisms that are evolved in microRNA-biology to drive cancer progression. Thereby, we also want to outlay potential novel therapeutic strategies. After providing a brief introduction to general miRNA biology and basic information about HCC and melanoma, this review depicts prominent examples of potent oncomiRs and tumor-suppressor miRNAs, which have been proven to drive diverse cancer types including melanoma and HCC. To develop and apply miRNA-based therapeutics for cancer treatment in the future, it is essential to understand how miRNA dysregulation evolves during malignant transformation. Therefore, we highlight important aspects such as genetic alterations, miRNA editing and transcriptional regulation based on concrete examples. Furthermore, we expand our illustration by focusing on miRNA-associated proteins as well as other regulators of miRNAs which could also provide therapeutic targets. Finally, design and delivery strategies of miRNA-associated therapeutic agents as well as potential drawbacks are discussed to address the question of how miRNAs might contribute to cancer therapy in the future.

## 1. The Emerging Role of miRNAs as Therapeutic Targets in Cancer

According to the last version of the human genome (GRCh38/hg38), the length of the human genome contains about 3.2 billion nucleotides but only about 20,000 protein-coding genes [1]. Thus, the major part of the human genome comprises a huge variety of non-coding RNAs, which are continuously attracting more and more interest of researchers. Many of these non-coding RNAs were considered as non-functional for a very long time. The discovery of RNA-interference (RNAi), a mechanism mediated by one specific family of those non-coding RNAs—so-called microRNAs (miRNAs, miRs)—was groundbreaking [2,3]. MiRNAs are involved in the regulation of all major cellular processes, including proliferation, apoptosis, cell-cycle regulation and differentiation [3,4,5,6,7,8]. Until today, more than 1800 miRNA sequences have been discovered in the human genome [9] and these were estimated to regulate ~50% of all human transcripts [10,11,12]. As a consequence, abnormalities in miRNA activity were found to strongly contribute to the formation and progression of many diseases including cancer [13,14,15]. During the last decade, more than 7000 patents related to miRNAs were granted in Europe and more than 12,000 in the USA [16]. More than half of these patents are based on miRNA- or siRNA-associated mechanisms in cancer development and progression. To date, the U.S. National Library of Medicine lists 856 clinical trials containing miRNAs [17].

Of note, miRNAs are stable in the serum [18] and can be applied as diagnostic and prognostic biomarkers [19,20,21,22]. Accordingly, more and more novel miRNAs are identified as crucial diagnostic and prognostic markers in all types of cancer such as oral cancer [23], glioblastoma [24], melanoma [25], liver cancer [26], colon cancer [27], gastric cancer [28], breast cancer [29], bladder cancer [30] and pancreatic cancer [31].

Likewise, they constitute promising therapeutic targets against cancer [32,33,34]. In this review, we want to focus on the emerging role of miRNAs as therapeutic targets in two specific cancer types—melanoma and hepatocellular carcinoma. Both cancer types show strong evidence for a significant implication of miRNAs in tumor development and progression [35,36,37,38,39,40,41]. By unraveling which common miRNAs and related pathways affect the development and progression of these—at first sight—dissimilar cancer types, one can learn that diverse cancer cells take advantage from similar and conserved mechanisms that have evolved in miRNA-biology.

## 2. Introduction to miRNA-Biology

**Figure 1:**
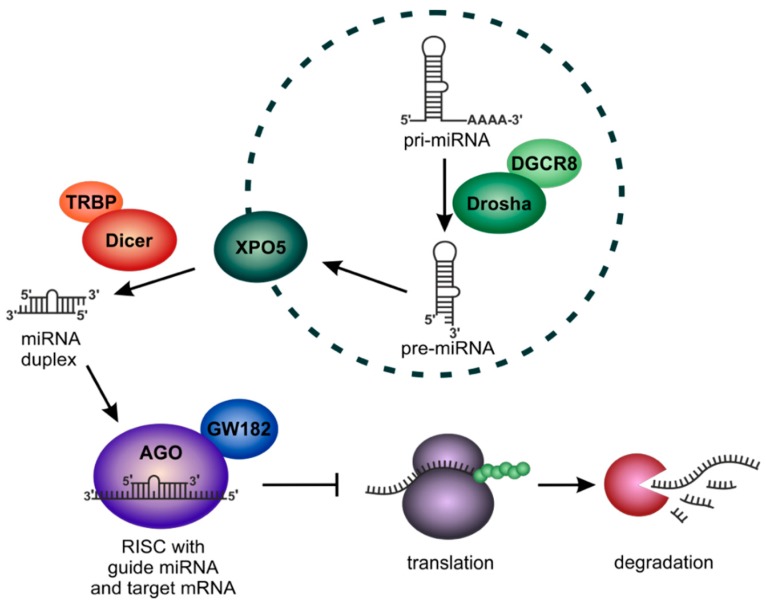
MiRNA processing pathway. Long primary miRNA transcripts (pri-miRNA) are processed in the nucleus by Drosha and DGCR8 [43,44,45,46]. The pre-miRNA is transferred into the cytoplasm by Exportin 5 (XPO5) and further processed by Dicer and TRBP [47,48]. The resulting miRNA duplex is loaded onto AGO at which point one strand is degraded [51,52]. The remaining mature miRNA strand forms the “RNA induced silencing complex” (RISC) together with AGO and GW182 [51,53]. The main function of the RISC is the translational repression of complementary target mRNAs [54].

Human miRNAs are transcribed in the cell nucleus as long primary transcripts containing a characteristic stem-loop structure of internally paired RNA bases (Figure 1) (for a detailed review on miRNA biogenesis see for example Reference [42]). Still in the nucleus, the primary miRNA transcript (pri-miRNA) is processed by the so-called microprocessor complex consisting of the enzymes Drosha and DiGeorge syndrome critical region 8 (DGCR8) [43,44,45,46]. The processed miRNA precursor (pre-miRNA) is translocated into the cytoplasm via the nuclear export factor Exportin-5 (XPO5) [47] and recognized by a second processing enzyme, Dicer [48], which cuts the pre-miRNA to a ~21–23 nucleotide double-stranded miRNA-Duplex [49]. The Dicer cofactor human immunodeficiency virus (HIV)-1 transactivating response RNA-binding protein (TRBP) recruits one of four human Argonaute proteins (AGO1-4) [50]. AGO binds to the miRNA and at the same time one miRNA strand is degraded [51,52]. The remaining strand represents the mature miRNA which is called the “guide strand.” Together with AGO, the mature miRNA forms the “RNA-induced silencing complex” (RISC) [51,53].

Subsequently, the mature miRNA guides the RISC to its target messenger RNA (mRNA) via complementary base pairing. For this interaction, the miRNA “seed” region comprising at least nucleotides 2–7 of the miRNA base pairs to the target mRNA [55]. Together with cofactors from the GW182 protein family, AGO mediates the translational repression of the target mRNA [54]. The repression occurs either at the translation initiation step via interfering with eukaryotic translation initiation factor eIF4E-binding to the mRNA 5′-cap-structure [56,57,58] and with ribosome recruitment [59] or at post-initiation steps [60,61,62,63]. Current models suggest that miRNA-mediated translational repression is further mediated by displacement of eIF4A1 or its paralogue eIF4A2 or by recruitment of the translational repressor and decapping activator DEAD box protein 6 (DDX6) [64,65]. However, the precise mechanism how DDX6 represses translation is unknown) [64].

In parallel to inhibition of translation, the AGO cofactors trinucleotide repeat containing 6 (TNRC6A, TNRC6B and TNRC6C), which belong to the GW182 protein family, can recruit cellular de-adenylation as well as the de-capping machinery and thereby initiate the decay of target mRNAs [65,66,67,68]. Indeed, decay of miRNA targets represents the dominant effect of miRNAs at steady state in cultured mammalian cells [64]. In more detail, degradation of miRNA targets is catalyzed by enzymes of the 5′-to-3′ mRNA decay pathway, which mediate de-adenylation, followed by de-capping and finally by degradation of mRNAs from the 5′ end. The activation of this pathway is possible because GW182 proteins bridge the interaction of AGO proteins and downstream effector complexes like the de-adenylation complexes PAN2-PAN3 and CCR4-NOT [67]. Here, GW182 proteins were shown to interact with their partner proteins by insertion of tryptophan residues into hydrophobic pockets which are exposed on the surface of AGO proteins as well as on the de-adenylation-associated proteins PAN3 and NOT9 [64].

Next to AGO-mediated translational repression and initiation of the deadenylation-decapping-degradation machinery, the AGO2 isoform additionally shows catalytic activity and can directly cleave target mRNAs if the miRNA (or siRNA) exhibits perfect complementarity to the target [69,70,71]. However, in mammalian cells, perfect miRNA-target complementarity is uncommon [64]. Together, the main function of the miRNA-pathway is the translational repression of specific target mRNAs.

## 3. The Role of miRNAs in Melanoma and Hepatocellular Carcinoma

Melanoma is a highly aggressive type of skin cancer. It reveals a high rate of metastasis and contributes to about 90% of skin cancer-related death [72]. Melanoma accounts for 5.5% from a total of 1,762,450 new cancer cases and for 1.2% of 606,880 estimated cancer-related deaths in the USA as estimated for the year 2019 by the American Cancer Society [73]. Moreover, the worldwide incidence rates of melanoma are still increasing [74]. The highest rate of newly occurring melanoma of 50–60 new cases per 100,000 inhabitants can be found in Australia [72]. Cutaneous melanoma derives from malignantly transformed melanocytes in the epidermis of the skin. Melanocytes are pigment-producing cells and deliver the pigment melanin to surrounding keratinocytes [75]. The most important function of melanin is protection from DNA damage caused by UV radiation and the absorption of radiation-induced radical ions and reactive oxygen species [76]. The main risk factor for the development of malignant melanoma is an episodically enhanced UV exposition [77,78], which is especially enforced in the last decades by the change in leisure habits like enhanced outdoor activities, sunbaths and shorter clothing. Thereby, particularly people with pale skin, red hair and freckles are at high risk, mostly bearing genetic variations of the melanocortin-1 receptor, which induces a sun-sensitive skin type [79]. Further risk factors for melanoma development are family predisposition [80] as well as multiple occurrences of melanocytic nevi (which are benign proliferations of melanocytes in the skin and can be transformed to precursor lesions of melanoma) [81]. In advanced/metastatic disease, systemic first-line therapeutic options are specific BRAF-inhibitors for BRAF^V600E^-mutated melanomas [82] as well as immune checkpoint inhibitors [83] but the understanding of emergence of acquired resistance to these therapies is still an unmet clinical need.

Many studies revealed that the expression of several miRNAs is deregulated in melanoma cells and that aberrant miRNA expression is undoubtedly linked to important processes affecting tumor formation and progression [35,36,84,85,86,87,88,89,90,91]. One example are members of the *let-7* miRNA family which are involved in melanoma invasiveness [92], cell cycle promotion [93] and metabolism [94]. Another example is *miR-137*, which regulates the expression of MITF in healthy melanocytes [95] and was the first miRNA described to be associated with melanoma development [96]. MiRNAs are not only differentially expressed between healthy melanocytes and transformed melanoma cells but can also reflect different melanoma subtypes related to varying genetic backgrounds [36,97]. Interestingly, we and other groups could show that a high number of miRNAs is upregulated in melanoma [36], which stands in contrast to many other tumor types, where miRNAs are mainly downregulated during tumor progression [98,99,100]. The reason for this melanoma-unique miRNA upregulation is still unclear.

Next to melanoma, the incidence and mortality rates of hepatocellular carcinoma (HCC) rise faster than for any other type of cancer worldwide. Liver cancer was estimated by the American Cancer Society to account for 2.4% of all new cancer cases in 2019 in the USA and for 5.2% of all cancer-related deaths [73]. In most cases, HCC develops as a consequence of underlying liver disease and is most often associated with liver cirrhosis. In North America and Europe, chronic inflammatory liver diseases are the major risk factors for the development of cirrhosis with subsequent HCC development. Most frequent causes are chronic infection with hepatic B and C viruses (HCV and HBV) and chronic alcohol abuse. Furthermore, so called non-alcoholic liver disease and steatohepatitis caused by obesity or other members of the metabolic syndrome are emerging as most frequent cause of cirrhosis and HCC, respectively, in developed countries [101]. HCC has a poor prognosis because it is often diagnosed at advanced stages. HCC is not amenable to standard chemotherapy and is resistant to radiotherapy. In early stages, surgical resection, local ablative procedures and liver transplantation are potentially curative treatment options. However, most patients are diagnosed at intermediate and advanced stages of the disease and the systemic treatment options for these patients include multi-kinase inhibitors, like sorafenib and lenvatinib, which show only a modest survival benefit [82,102].

Studies using a combination of “omics” technologies, miRNA studies, combinatorial chemistry and bioinformatics have recently provided novel insights into the gene expression and protein profiles during different stages of HCC [101]. MiRNAs can modulate various physiological as well as pathological mechanisms in liver biology, including development and progression of HCC [103]. Aberrant miRNA expression correlates with severity and prognosis of HCC [104]. For example, *miR-122* is downregulated in HCC and represents an attractive treatment option to sensitize HCC cells to standard systemic therapeutic agents such as sorafenib [105]. Another study revealed that in HCC with cirrhotic background, members of the *let-7* miRNA-family, *miR-22-1* and *miR-145* were downregulated [106]. In these tissues, *miR-122* was also downregulated and its target gene product cyclin G1 was highly expressed and promoted growth of HCC cells [106]. *MiR-122 *re-expression significantly reduced in vitro migration, invasion and anchorage-independent growth of HCC cells. Furthermore, *miR-122* re-expression reduced in vivo tumorigenesis, angiogenesis and intrahepatic metastasis in an orthotopic liver cancer model [107]. Many further examples of dysregulated miRNAs including the strong tumor-suppressor *miR-622* have been proven to affect critical mechanisms in HCC progression [108,109], thereby outlining the potentially major impact of miRs as therapeutic (liver) cancer targets.

Although HCC and melanoma are highly malignant cancer types deriving from completely different origins and having different types of risk factors, their regulation by similar miRNAs (see above, for example, *miR-622*, *let-7*) highlights the ubiquitous involvement of miRNAs (and related pathways) in cancer biology. Therefore, some of the most prominent miRNAs involved in melanoma and HCC are highlighted in more detail in the following sections.

## 4. Specific miRNAs as Therapeutic Agents in Melanoma and HCC—A Focus on Target Genes

Numerous studies have described so-called “miRNA signatures” associated with specific biological functions, including cancer development and progression [13,85,98,99,100,110,111,112]. Since one miRNA can regulate up to hundreds of different target genes in a cell [69,113,114], the administration of single miRNAs as therapeutic targets raises the problem of a potentially widespread functional heterogeneity of one miRNA in different tumors types and potential adverse side effects to normal tissue [115,116]. Therefore, research addressing miRNAs as therapeutic targets should focus on miRNAs that majorly or desirably act solely as tumor-suppressors or oncogenes in one specific setting to avoid mutual neutralization effects. A tumor-suppressive or oncogenic function of one miRNA depends on the set of regulated target genes and affected signaling pathways. In the following, we want to focus on prominent examples of miRNAs that have been proven to be “specific” tumor-suppressors or oncogenes, respectively, in two exemplary types of typical miRNA-regulated cancers, melanoma and HCC. These features qualify the here described examples of miRNAs for potentially specific and highly potent miRNA-based therapeutic strategies.

## 5. Tumor-Suppressor miRNAs in Melanoma and HCC

### 5.1. The Let-7 miRNA Family

One of the first miRNAs that was shown to be strongly associated with cancer development was *let-7*, regulating the expression of the potent oncogene rat sarcoma (RAS) [117]. RAS proteins including the isoforms KRAS and NRAS are amongst the most prominent oncogenes and were recently described to play major roles also in melanoma [5,118] and HCC [109,119,120]. Let-7 represents a highly conserved family of miRNAs [121]. In humans, ten mature *let-7* miRNA family members were described, encoded by 13 genomic regions [122]. Let-7 was also shown to play a pivotal role during embryogenesis [123].

**Figure 2:**
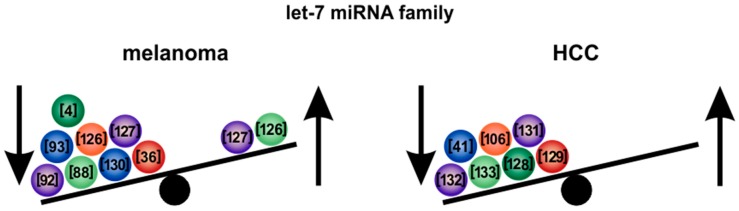
*Let-7* members are strongly downregulated and function as potent tumor-suppressors in melanoma and hepatocellular carcinoma (HCC). References (numbers in brackets indicate according references of studies) showing differential expression (indicated by arrows) of single *let-7* family members in melanoma and HCC.

Members of the *let-7* family downregulate the expression of embryonic genes during late embryonic development, which may not be expressed in the adult, for example, the embryonic gene high mobility group A2 (HMGA2) [124]. The expression of HMGA2 is reactivated during early cancer development, indicating that tumor formation appears as a reversion of embryogenesis [124]. Let-7 family members are important players during this process. In cancer, *let-7* members function as potent tumor-suppressive miRNAs, which are predominantly downregulated during tumor progression [125]. Let-7 family members are also strongly involved in both melanoma [4,88,92,93,126,127] and HCC [41,106,128,129] (Figure 2, Table 1).

**Figure 3:**
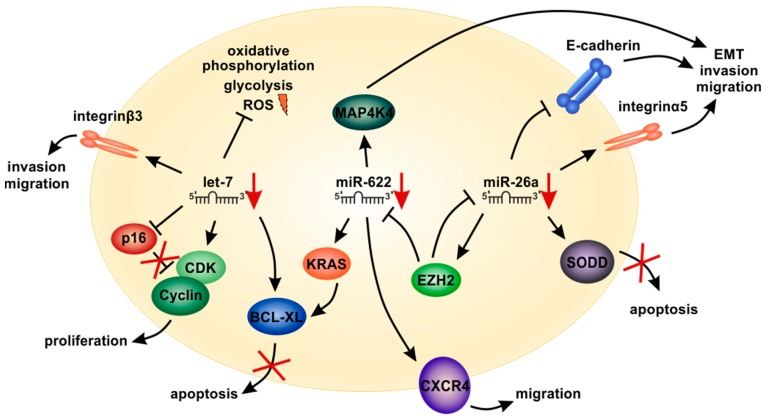
Important tumor suppressive miRNAs and their impact on cancer cells. MiRNAs *let-7*, *miR-622* and *miR-26a* are downregulated during tumor development in both melanoma and HCC (and also many other cancer types) (indicated by red arrows), thereby influencing major target genes and according cellular pathways. Downregulation of *let-7* induces de-repression of integrin β3 promoting cancer cell migration and invasion [92]. It further releases cell cycle promoting cyclins and CDKs [93] and inhibits the cell cycle inhibitor p16 [133]. Low expression of *let-7* interferes with apoptosis via induction of the antiapoptotic protein BCL-XL [132]. Furthermore, cancer associated downregulation of *let-7* results in reduced oxidative phosphorylation, glycolysis and production of ROS [94]. Downregulation of *miR-622* results in an increase of its target KRAS [5,109]. KRAS can also interfere with the apoptosis pathway via upregulation of BCL-XL [109]. *MiR-622* downregulation also unreleases its target CXCR4 which mediates migration of tumor cells [141]. Further, low *miR-622* expression induces de-repression of MAP4K4 promoting epithelial to mesenchymal transition (EMT) and invasiveness [142,143]. Low levels of *miR-26a* in tumor cells lead to increased integrin α5 expression and reduced E-cadherin expression inducing EMT [144,145,146]. It further induces the release of anti-apoptotic SODD [147]. Moreover, both *mir-622* and *miR-26a* are suppressed by EZH2 in tumor cells [141,148,149]. Simultaneously, decreased *miR-26a* expression releases its target EZH2, creating a regulatory feedback loop [148,149,150,151].

In melanoma, it has been shown that experimental overexpression of *let-7a* interferes with cancer cell invasiveness via downregulation of integrin β3 [92] (Figure 3). Overexpression of **let-7b** in melanoma cells also reduced the expression of the cell cycle promoters cyclin D1, cyclin D3, cyclin A and cyclin-dependent kinase 4 (CDK4) [93]. Furthermore, it reduced cell growth, increased expression of anabolism-associated proteins [94] and enhanced oxidative phosphorylation and glycolysis, leading to elevated reactive oxygen species (ROS) formation [94] (Figure 3).

During development of HCC, *let-7*-family members were shown to be differentially expressed. Expression levels of *let-7a*, *b* and *c* were upregulated in non-tumorous liver diseases, including chronic hepatitis and liver cirrhosis [128]. During early stages of HCC, however, *let-7a*, *b* and *c* were significantly downregulated as compared to the non-tumorous liver tissue [128]. This points to potential tumor-suppressive functions that are lost during cancer development. Overexpression of *let-7a* in HCC cells decreased cell viability and promoted an epithelial-like phenotype, which decreased sphere formation and prohibited the self-renewal ability of HCC stem-like cells by affecting the Wnt signaling pathway [134]. Furthermore, overexpression of *let-7a* improved sensitivity to cetuximab in HCC cells, which was mediated by *let-7*-induced inhibition of STAT3 [135]. In addition, overexpression of *let-7g* decreased proliferation of HCC cells by affecting the expression of oncogenic c-Myc and upregulation of tumor-suppressive p16 [133] (Figure 3).

Interestingly, the mRNA of the hepatitis B virus was also proven to be a target gene of *let-7g* [136]. Infection with HBV interfered with *let-7g* function, thereby facilitating liver cancer growth [136]. Overexpression of *let-7g* and *let-7i* likewise decreased HCC cell proliferation and promoted apoptosis via repression of the antiapoptotic protein BCL-XL, which was synergistically regulated by the two miRNAs [132] (Figure 3). Regulation of BCL-XL by **let-7c** and *let-7g* was furthermore shown to enhance apoptosis in response to sorafenib treatment [129].

Next to melanoma and HCC, the *let-7* family of miRNAs was also reported to be differentially regulated and/or to reveal prognostic, diagnostic or functional roles in many other cancer types, like uveal melanoma [137], neuroendocrine tumors [138], neuroblastoma [139] and colorectal cancer [140].

In summary, the *let-7*-family of miRNAs consists of the most potent and most widely investigated tumor-suppressive miRNAs in diverse cancer types, including melanoma and HCC. Considering its potent function in stem cell biology and embryology, it appears that *let-7* functions as a principal gatekeeper in cancer development and represents a promising tool for combination with chemotherapeutic treatment in HCC and melanoma.

### 5.2. MicroRNA-622

*MiR-622* is quite unexplored and was first described in the year 2010 to play a role in colon cancer, when nasopharyngeal carcinoma-associated gene 6 (NGX6) was shown to be a novel putative tumor-suppressor gene able to regulate the expression of several miRNAs including *miR-622* [152]. Du et al. described *miR-622* as one of two novel miRNA families expanded in the human genome, which are mostly embedded in or close to proteins with conserved functions [153]. During the first years after its exploration, the detailed function of *miR-622* concerning particular tumor entities remained largely unclear, as data on its function either as oncogene or tumor-suppressor were controversial—In 2011, Guo et al. found *miR-622* to be down-regulated in gastric cancer, where it could promote invasion, tumorigenesis and metastasis of gastric cancer cells both, in vitro and in vivo. Furthermore, ING1 was shown to be a direct target of *miR-622* [154]. In 2014, Xie et al. confirmed that *miR-622* is downregulated in gastric cancer [155]. Moreover, *miR-622* was overexpressed in Taxol-resistant ovarian cancer cells and was shown to be able to serve as a significant prognosis marker of the chemo-resistant patient group. Downregulation of *miR-622* was associated with better survival, perhaps increasing the sensitivity of cancer cells to Taxol [156]. Odenthal et al. also described *miR-622* to be dysregulated in esophageal cancer [157]. Altered expression of *miR-622* was also shown in pancreatic and ampullary adenocarcinoma [158].

**Figure 4:**
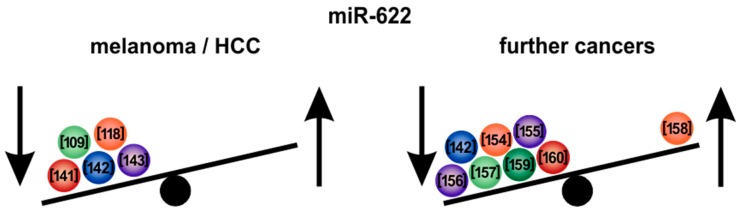
*MiR-622* is a strongly downregulated tumor-suppressive miRNA in melanoma, HCC and also in other cancer types. Several studies (numbers in brackets indicate according references) showed differential expression (indicated by arrows) of *miR-622* in melanoma, HCC and other further cancer types.

However, in recent years, it became more and more evident that *miR-622* is one of the most potent tumor-suppressor miRNAs (Figure 4). *MiR-622* was amongst 13 miRNAs that were shown to be strongly associated with pathological complete response to neoadjuvant chemoradiotherapy in rectal cancer patients [159]. Moreover, *miR-622* was described as one of two most differentially expressed miRNAs between sporadic colon cancer and colon cancers with microsatellite instability [160]. Several studies suggested that *miR-622* could affect proliferation, clonogenicity and migration in cancer cells by distinct pathways [142,161]. Recently, we identified wildtype KRAS as a novel therapeutic target in melanoma and showed that KRAS inhibition functions synergistically with BRAF inhibition [118]. Several miRNAs have been described recently as emerging and crucial KRAS regulators in different cancer types [162,163]. In another study, KRAS was shown by our group to be majorly regulated by *miR-622* in melanoma [5]. Furthermore, acquired resistance to BRAF inhibitors in melanoma was dependent on dynamic regulation of KRAS expression and could be overcome by KRAS inhibition. This highlights the strong and potential therapeutic impact of the *miR-622*-KRAS-axis in melanoma [5,118].

Interestingly, in HCC, we also found increased wild-type KRAS expression in HCC compared to non-tumorous liver which correlated with tumor size, proliferation and poor survival of patients [109]. Using bioinformatic analyses and reporter assays, we identified *miR-622* as a direct regulator of KRAS in HCC. Like in melanoma, *miR-622* expression was strongly downregulated and inversely correlated with KRAS expression in human HCC tissues. Thus, targeting wild-type KRAS might represent a promising therapeutic strategy to enhance treatment response in both HCC and melanoma. In this respect, we showed that deltarasin—a novel small-molecule KRAS inhibitor—strongly inhibited proliferation and induced apoptosis in HCC and in melanoma cells, which was associated with the inhibition of the downstream RAF/MAPK- and PI3K/AKT pathway as well as with the down-regulation of anti-apoptotic (BCL-2, BCL-XL) and the up-regulation of pro-apoptotic (BAX, PUMA) molecules [109] (Figure 3). Affection of apoptosis-related proteins including BCL-XL also resembled the functions of *let-7* [129,132], pointing to co-regulation of major cancer-pathways by diverse tumor-suppressor miRNAs (Figure 3).

The anti-tumor effects of deltarasin were also validated and confirmed in vivo applying an orthotopic HCC mouse model and KRAS inhibition by deltarasin markedly enhanced sorafenib-induced tumor cell apoptosis and inhibition of proliferation in HCC cells [109]. Interestingly, sorafenib treatment caused a dose-dependent up-regulation of KRAS in HCC cells which was associated with the development of sorafenib resistance. Importantly, KRAS inhibition could re-sensitize these cells for sorafenib-induced toxicity [109,164]. Therefore, the design of clinical trials in HCC patients evaluating novel KRAS-inhibiting drugs alone or in combination with sorafenib in second-line/third-line treatment was proposed to address a currently unmet medical need [164]. According to our findings, other wild-type isoforms of MAPK-pathway-associated players are just beginning to be recognized as potent therapeutic targets in cancer. For instance, it is now known that elevation of wild-type RAF expression or enhanced RAS activity could lead to drug resistance in mutant BRAF tumors [165]. Notably, melanoma is a typical BRAF-mutated cancer type. Therefore, it is of importance that also in melanoma the *miR-622*-target KRAS [5] was shown by our group to strongly affect BRAF-inhibitor resistance [118]. This strongly resembled our findings in HCC and thus points to common and crucial cancer-pathways regulated by miRNAs in different cancer types. In contrast to proliferation and apoptosis, *miR-622*’s inhibitory effect on the migratory activity of HCC cells was independent of KRAS-suppression [109]. These data are in line with two recent studies that described further tumor-suppressive functions of *miR-622* in HCC. Liu et al. identified *miR-622* as negative regulator of CXC chemokine receptor 4 (CXCR4) in HCC and showed that the inhibitory effect of *miR-622* on migration of HCC cells strongly depends on CXCR4 suppression [141]. In contrast and according to our findings on *miR-622*-mediated KRAS suppression which reduced proliferation, the growth-suppressive effects of *miR-622* on HCC cells were only minimally affected by its effect on CXCR4 expression [141]. Song et al. found that *miR-622* negatively regulates mitogen-activated protein 4 kinase 4 (MAP4K4) in HCC but overexpression of MAP4K4 only partially reversed the growth-suppressive effects of *miR-622* on HCC cells [142]. In a recent study, the same group also demonstrated that MAP4K4 promoted the epithelial-mesenchymal transition and invasiveness of HCC cells largely via activation of the c-Jun N-terminal kinase(JNK) and the nuclear factor ‘‘kappa-light-chain-enhancer’’ of activated B-cells (NF-κB) signaling [143].

In summary, *miR-622* exhibits potent tumor-suppressive functions in HCC and in melanoma via affection of several relevant target genes and mechanisms, respectively, with KRAS being the major target responsible for *miR-622*’s inhibitory effect on HCC proliferation and clonogenicity [5,109,118]. Potentially, *miR-622* serum levels might be used as a predictive marker for HCC and melanoma (progression). However, detection of strongly downregulated miRNAs would be technically demanding, while quantification of increased serum-miRNAs could indeed serve as reproducible biomarkers [166].

### 5.3. MicroRNA-26a

**Figure 5:**
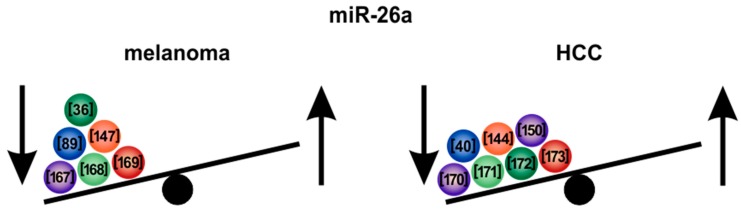
Downregulation of tumor-suppressor *miR-26a* in melanoma and HCC. Several studies (numbers in brackets indicate according references) showed differential expression of *miR-26a* in melanoma and HCC.

Another potent tumor-suppressive miRNA is the *miR-26a*, which is strongly downregulated in both melanoma [36,89,147,167,168,169] and HCC [40,144,150,170,171,172,173] (Figure 5). In melanoma, re-expression of *miR-26a* induced cell cycle arrest and increased apoptosis [167,174]. This phenotype was mediated via downregulation of the anti-apoptotic silencer of death domains (SODD) protein [147]. The potential therapeutic use of this mechanism has already been discussed previously [19]. Furthermore, mouse melanoma cells transfected with *miR-26a* showed significantly reduced tumor growth in vivo [174]. Qian et al. described that *miR-26a* targets the microphthalmia-associated transcription factor (MITF), a key regulator of melanoma development [174]. Thus, *miR-26a*, which has widely been demonstrated to be involved in key tumorigenic processes also represents an interesting target for melanoma therapy.

In HCC, re-expression of *miR-26a* inhibited proliferation, migration and invasion [170]. *MiR-26a* was shown to target DNA methyltransferase 3 beta (DNMT3B), which is frequently upregulated in HCC tissues [170]. Zhao et al. recently showed that *miR-26a* re-expression in HCC reduced cell proliferation both in vitro and in a xenograft model [150]. However, in the same study, *miR-26a* promoted HCC tumor cell migration, invasion and metastasis in vivo after injection of tumor cells into the tail vein of nude mice, probably by downregulation of phosphatase and tensin homolog (PTEN) [150]. Other studies also showed that a low amount of *miR-26a* in HCC leads to activation of the Wnt/β-catenin pathway, reduced E-cadherin expression and induction of epithelial to mesenchymal transition (EMT) [144,145]. Therefore, in contrast to early cancer development, *miR-26a* might also have oncogenic functions in advanced tumor stages and metastasis in HCC and other types of cancer (Figure 5).

A further potent oncogenic target gene of *miR-26a* in HCC is the enhancer of zeste homolog 2 (EZH2) [148,149,150,151]. Vice versa, EZH2 can suppress *miR-26a* expression via trimethylation of H3K27 in the *miR-26a* promoter creating a negative feedback loop that is imbalanced in HCC cells [148,149]. Interestingly, *miR-622* expression can also be regulated by EZH2 [141] indicating mutual/synergistic regulation of *miR-622* and *miR-26a* in HCC.

Gao et al. found that p53 mediated activation of *miR-26a* induced apoptosis in HCC cells [175]. Furthermore, low expression of *miR-26a* correlated with a poor prognosis of HCC patients [144,176]. This finding was also confirmed in patients with HBV-induced HCC [177]. *MiR-26a* was also associated with resistance to the chemotherapeutic drug doxorubicin [173].

Further important and validated target genes of *miR-26a* in HCC are GSK3β [145], the E3 ubiquitin ligase F-box protein 11 [171], the sialyltransferase ST3GAL6 [178], the fucosyltransferase FUT8 [179], integrin α5 [146], the hepatocyte growth factor [180], interleukin-6 [181], the estrogen receptor-α [182] and the cyclin-dependent kinase 6 as well as cyclin E1 [183]. All those proteins are involved in promoting HCC tumor initiation and progression, making this miRNA an interesting target option for HCC therapy.

Moreover, next to melanoma and HCC, *miR-26a* has also been reported to play potential crucial roles in diverse further cancer types including bladder cancer [184], osteosarcoma [185], multiple myeloma [186], thyroid carcinoma [187], pancreatic cancer [188] and colorectal cancer [189].

Together, next to *let-7* and *miR-622*, *miR-26a* represents a third potent tumor-suppressive miRNA affecting diverse cancer-related hallmarks in different cancer types. Therefore, *miR-26a* has the potential to become a further promising target for future therapeutic approaches.

The three examples of *let-7*, *miR-622* and *miR-26a* clearly show that some of the most prominent miRNAs are downregulated, have tumor-suppressive functions and affect chemoresistance and survival in not only one specific but in diverse cancer types. This underlines the conserved biological functions of these three miRNAs in cancer. Moreover, comparing known target genes of such miRNAs, one can find that these tumor-suppressive miRNAs also share similar pathways that emerged as major and promising therapeutic targets in cancer therapy (Figure 3). We analyzed the seed sequences of those important tumor-suppressive miRNAs and surprisingly, there were no significant overlaps (data not shown). Thus, similar regulation of target genes by these three exemplary miRNAs besides seed homology emphasizes the importance of an efficient regulation of the described target genes for tumor development.

## 6. OncomiRs

### 6.1. MicroRNA-221

**Figure 6:**
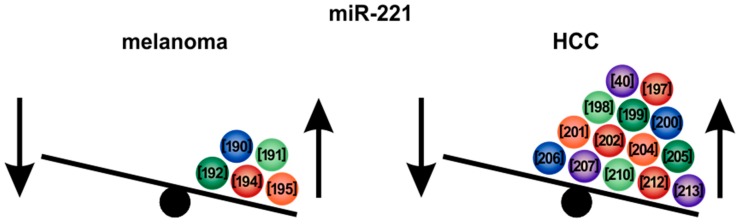
Upregulation of OncomiR *miR-221* in melanoma and HCC. References (numbers in brackets indicate according references of studies) showing differential expression (indicated by arrows) of *miR-221* in melanoma and HCC.

**Figure 7:**
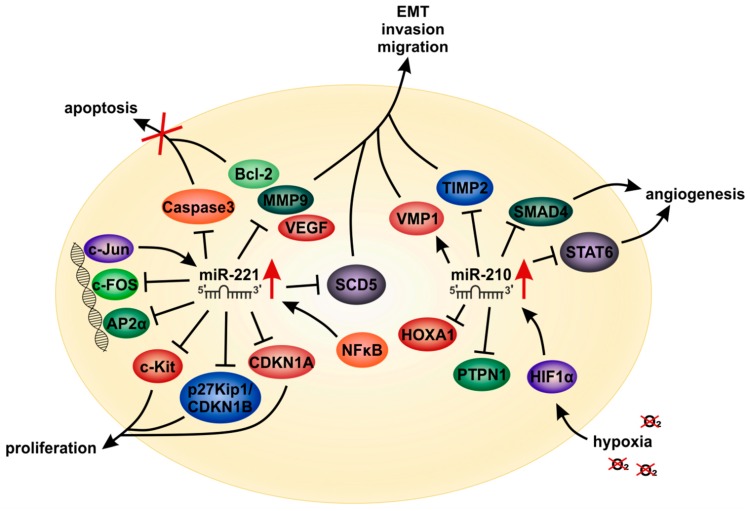
OncomiRs *miR-221* and *miR-210* and their impact on cancer cells. The miRNAs *miR-221* and *miR-210* are significantly upregulated during tumor development of melanoma and HCC (indicated by red arrows) which leads to interference with important cellular pathways. *MiR-221* downregulates the transcription factors c-FOS [196] and AP2α [191] and is regulated itself by c-Jun and the NFκB pathway [213]. NFκB regulation also leads to suppression of the *miR-221* downstream genes Bcl-2, VEGF and MMP-9 thus inhibiting apoptosis [196]. *MiR-221*-associated anti-apoptotic activity is further mediated by targeting caspase-3 [209]. Regulation of Bcl-2, VEGF and MMP-9 by *miR-221* can also induce an invasive phenotype which is further mediated by *miR-221* suppressing SCD5 and thereby promoting EMT [193]. Additional *miR-221* targets are c-Kit, p27Kip1/CDKN1B and CDKN1A whose downregulation in cancer induces cell proliferation [194,195,212]. *MiR-210* can also influence EMT and migration via inhibition of TIMP2 [215] and activation of VMP1 [216]. Downregulation of *SMAD4* and *STAT6* by *miR-210* promotes angiogenesis [217]. Further important targets of *miR-210* in tumor cells are HOX1A and PTPN1 interfering with the immune response [218]. *MiR-210* expression is induced during hypoxia [219,220] through regulation by HIF1α [221].

*MiR-221* expression is significantly enhanced in melanoma compared to melanocytes and healthy tissues and further increases when melanoma cells gain metastatic features [190,191] (Figure 6). Due to high *miR-221* levels in patient sera, which were shown to correlate with tumor stages (i.e., thickness/infiltration), this miRNA might serve as a diagnostic and prognostic biomarker for melanoma [190,192]. *MiR-221* targets (together with the highly homologous *miR-222*) the stearoyl-CoA desaturase (SCD5), thereby inducing its degradation which is associated with an epithelial-to-mesenchymal (EMT) phenotype during melanoma progression [193] (Figure 7). Furthermore, *miR-221* can facilitate cell cycle progression and proliferation via down-regulation of the tumor-suppressor p27Kip1/CDKN1B and the receptor tyrosine kinase c-KIT, thereby promoting melanoma progression both in vitro and in vivo [194,195]. Moreover, together with *miR-222*, *miR-221* can downregulate the transcription factor AP2α, which is commonly lost in advanced melanoma [191]. A further target of *miR-221* in melanoma is the AP-1 family transcription factor c-FOS [196].

In HCC, *miR-221* was also described in numerous studies to be a striking example of a highly potent oncogenic miRNA (Figure 6). *MiR-221* levels are enhanced in HCC tissues, HCC cell lines and in the serum of HCC patients [40,197,198,199]. Therefore, likewise as in melanoma, *miR-221* could also serve as a biomarker for the diagnosis of HCC [200]. Moreover, chronic HBV or HCV infections have been shown to induce *miR-221* expression in hepatocytes [201,202]. Overexpression of *miR-221* in hepatocytes enhanced cell proliferation due to a rapid S-phase entry and supported liver regeneration [203]. High expression of *miR-221* in HCC patients also correlates with a poor survival [197,204]. It has been shown that *miR-221* can promote EMT [205] as well as HCC cell migration [206]. Accordingly, high expression of *miR-221* correlates with HCC lymph node metastasis [207]. *MiR-221* was shown to be released via extracellular vesicles by HCC cells, thereby inducing the activation of hepatic stellate cells [208]. Hepatic stellate cells, in turn, can promote a pro-metastatic environment for HCC cells [208]. High *miR-221* expression was further associated with sorafenib resistance in mouse and rat models of experimental HCC [209]. Fornari et al. identified caspase-3 as a target gene of *miR-221*, causing a *miR-221*-associated anti-apoptotic activity [209] (Figure 7). A further important target gene of *miR-221* was shown to be the cell-cycle regulator p27(Kip1) [210,211]. Moreover, *miR-221* targets are the E2F transcription factor 1 (*E2F1*), the phosphatase and tensin homolog (*PTEN*) and the cyclin-dependent kinase inhibitor 1 (*CDKN1A*), all belonging to critical cancer related pathways in HCC as well as other types of cancer including melanoma [212]. Bae et al. showed that a *miR-221* mediated suppression of HDAC6 was initiated by the JNK/c-Jun signaling pathway and by NFκBp65 nuclear translocation [213]. The phosphorylation of 4EBP1, which is a downstream effector of the PI3K-AKT-mTOR pathway, is also induced by *miR-221* [214], showing that *miR-221* influences several major cancerogenic pathways in cancer cells.

Treatment with anti-*miR-221* oligonucleotides has been shown to reduce development and malignant progression of liver nodules after experimental induction of chronic liver damage in mice [222]. Furthermore, anti-*miR-221* inhibited growth and invasion of HCC cells and induced apoptosis in an NFκB-mediated manner, as this signaling pathway is downregulated and the expression of downstream genes such as Bcl-2, VEGF and MMP-9 is inhibited [223].

Apart from melanoma and HCC, *miR-221* was reported to be critically involved also in different cancer types, including cervical cancer [224], retinoblastoma [225], breast cancer [226], colorectal cancer [227] and gastric cancer [228].

In summary, *miR-221* can be considered as one of the most potent oncogenic target miRNAs with major impact on melanoma and HCC progression and chemoresistance as well as crucial roles in further cancer types. Therefore and because of its pleiotropic and synergistic cancerogenic effects, targeting *miR-221* represents a desirable approach for futures cancer therapeutic strategies.

### 6.2. MicroRNA-210

**Figure 8:**
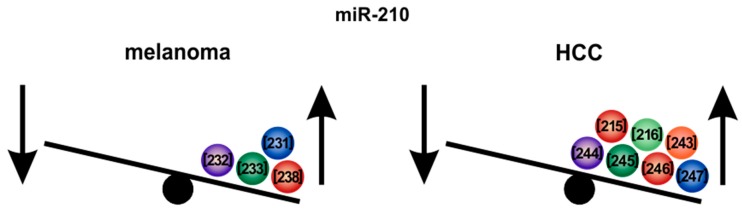
*MiR-210* is strongly upregulated and has oncogenic function in melanoma and HCC. References (numbers in brackets indicate according references of studies) showing differential expression (indicated by arrows) of *miR-210* in melanoma and HCC.

*MiR-210* represents a further example of a potent oncogenic miRNA in melanoma as well as in HCC (Figure 8). *MiR-210* expression is induced during hypoxia [219,220], a state which can often be found in solid tumors and which is associated with poor prognosis and resistance to radiation therapy [229]. Cancer cells have adapted to low oxygen availability and use the hypoxia-associated reprogramming to survive and to proliferate. *MiR-210* is an intronic miRNA which is encoded within a long non-coding transcript that contains a hypoxia inducible factor (HIF) response element [221]. HIF1α is the master regulator of hypoxia, which promotes an invasive phenotype [230]. Notably, HIF1α upregulates *miR-210* expression in melanoma [231] (Figure 7). *MiR-210* is significantly enhanced in melanoma cell lines as compared with melanocytes and in patient-derived tumor samples as compared with melanocytic nevi [232]. In patient samples derived from metastatic melanomas, *miR-210* expression was significantly elevated compared to nonmetastatic tumors [233]. Exosomes containing *miR-210* are secreted by melanoma cells and can be taken up by surrounding fibroblasts [234]. This causes an increase in aerobic glycolysis and a decrease in oxidative phosphorylation in the fibroblasts, where *miR-210* plays a pivotal role [234]. The metabolic reprogramming of tumor surrounding fibroblasts increases extracellular acidification and may build a pro-metastatic environment [234,235,236]. The small molecule methyl sulfone, has been shown to normalize the pro-metastatic metabolism of hypoxic melanoma cells via downregulating the expression of HIF-1α and, amongst others, simultaneously also reducing *miR-210* expression [237]. In a melanoma cell-derived xenograft mouse model, *miR-210* is overexpressed and inhibition of *miR-210* reduced the sensitivity of the tumors to MEK1/2 inhibition [238]. Additional important target genes of *miR-210* in hypoxic cells were shown to be PTPN1, HOXA1 and TP53I11 - downregulation of these genes interfered with the susceptibility of melanoma tumors to lysis by cytotoxic T-cells [218]. Furthermore, *miR-210* can enhance the immunosuppressive activity of tumor-surrounding myeloid-derived suppressor cells against T-cells thereby promoting tumor growth [239]. Therefore, *miR-210* could majorly influence immunotherapeutic strategies in melanoma, which were shown to be successful in recent years [240,241,242].

Likewise, *miR-210* was found to be significantly increased in HCC tissues [243] as well as in the serum of patients. Furthermore, *miR-210* was described to represent one of the most promising miRNA biomarkers for HCC [244,245]. High *miR-210* expression correlates with poor tumor-free and overall survival of HCC patients [243,245,246]. In addition, *miR-210* can be used to discriminate HCC from other metastatic malignancies in the liver [247]. Moreover, *miR-210* expression correlates in HCC with elevated tumor stages, vascular invasion and venous metastases indicating that *miR-210* could promote metastasis of HCC [216], similarly as described in melanoma. *MiR-210* is secreted by HCC cells in exosomes and high serum levels of *miR-210* are associated with higher microvessel density in vivo as well as with an improved angiogenesis in in vitro-assays [217,243]. This pro-angiogenic effect can be mediated by inhibition of the *miR-210* target genes *SMAD4* and *STAT6* in surrounding endothelial cells [217] (Figure 7). Resembling the above described findings in melanoma, *miR-210* expression was shown to be associated with a hypoxic tumor environment in HCC. In hypoxic conditions, *miR-210* is regulated by HIF1α and HIF3α and can promote metastasis of HCC cells via inhibition of tissue inhibitor of metalloproteinases 2 (TIMP2). Thereby *miR-210* is inducing an aggressive behavior of HCC cells and high *miR-210* levels correlate with a poor patient outcome [215]. Hypoxia-induced HCC cell metastasis can also be mediated by downregulation of vacuole membrane protein 1 (VMP1), which is a direct target of *miR-210* [216].

Apart from melanoma and HCC (e.g., in pancreatic cancer [248], breast cancer [249] and oral squamous carcinoma [250]), *miR-210* was also revealed as a promising diagnostic, prognostic or functional target, respectively. However, its definite role as either oncogene or tumor suppressor is not completely consistent in these cancers.

In summary, *miR-210* constitutes a promising target for tumor progression and invasiveness both in melanoma and HCC and was also shown to be involved in further cancer types.

## 7. How does miRNA Dysregulation Evolve?

### 7.1. Genetic Alterations, Transcriptional Regulation and miRNA-Editing

To use miRNAs as therapeutic targets, a detailed understanding of the precise mechanisms of how deregulation of miRNA expression and function in tumor cells occurs is essential. Like other deregulated genes, upregulation or suppression of miRNAs, respectively, is often a result of cancer-associated mutations or further genetic changes. Many miRNA genes are located in chromosomal regions, which are known as fragile in terms of frequent mutations, amplifications or chromosomal loss [251]. A single nucleotide polymorphism (rs10877887) in the promoter region of miRNA *let-7* is often found in HCC and was assumed to increase the risk of tumor development [252]. In melanoma, the examination of the gene locus 1p22, which often harbors inactivating mutations [253], led to the discovery of *miR-137*.

Furthermore, numerous mutations were found in the 3′-UTR regions of tumor-associated genes, thereby suppressing the binding of regulatory miRNAs [254]. On the other hand, mutations in one of the miRNAs strands can inhibit recognition of target mRNAs or can lead to an aberrant passenger to guide strand relation, which causes binding of alternative tumor-associated targets [53].

**Figure 9:**
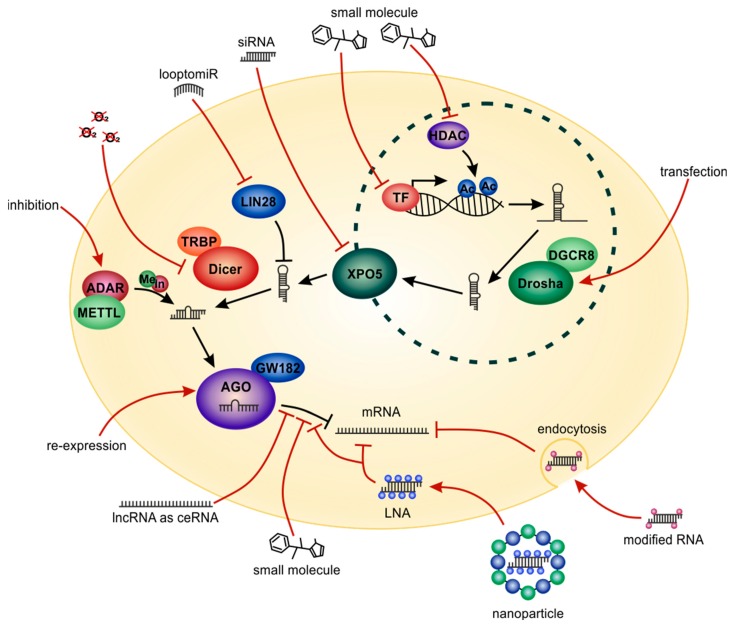
Potential ways to therapeutically target miRNAs and miRNA-related enzymes. Red arrows indicate multiple approaches for targeting miRNAs for therapeutic purposes. Small molecule targeting of epigenetic enzymes, for example, histone deacetylases (HDACs) or specific transcription factors (TF) can reactivate the expression of tumor-suppressive miRNAs [196,258,266,270]. Drosha expression could be induced or *XPO5* expression could be inhibited by siRNA leading to induction or repression of tumorigenic miRNAs [271,272]. To inhibit binding of the negative regulator LIN28 to the tumor-suppressive miRNA *let-7*, short, loop-targeting “looptomiRs” can be used [273]. Targeting Dicer could be a potentially promising approach for specific tumor conditions such as hypoxia [274]. MiRNA modifying enzymes, such as ADAR or METTL, could also be approached by therapeutic strategies [261,262,263,264]. AGO is strongly downregulated in melanoma and re-expression could represent a therapeutic option [275,276]. The inhibitory effect of tumor-specific miRNAs on their target mRNAs could be inhibited by sequestering the miRNAs using, for example, lncRNAs as competing endogenous RNAs (ceRNAs) [277,278,279], by small-molecule inhibitors [280] or modified oligoribonucleotides (e.g., LNAs) [281]. Those can be specifically delivered into tumor cells using a nanoparticle based system [282]. Modified RNA molecules can also be taken up via endocytosis [33].

Besides genetic variations, epigenetic changes or post-transcriptional modifications of miRNAs can lead to deregulated expression in tumor cells [255]. For example, DNA hypermethylation can initiate the downregulation of *miR-211* in melanoma tissue, which is a tumor-suppressive miRNA and suppressed in melanoma [256]. In HCC, numerous tumor-suppressive miRNAs including *miR-1*, *miR-124* and *miR-203* are downregulated during hepatocarcinogenesis as a result of promoter hypermethylation [257]. Targeting histone deacetylases (HDACs) by specific small molecule inhibitors may reactivate the expression of those tumor-suppressive miRNAs and could represent a promising therapeutic strategy [258] (Figure 9). We could show that the HDAC inhibitors suberanilohydroxamic acid (SAHA) and trichostatin A (TSA) showed promising results affecting proliferation, clonogenicity and the migratory potential of HCC cells in vitro and could also enhance the effects of sorafenib [259]. The HDAC inhibitors belinostat (as a monotherapy) and resminostat (in combination with sorafenib) were tested for HCC treatment in Phase I/II clinical studies and revealed promising results regarding drug response and patient survival [260].

Adenosine deaminase acting on RNA (ADAR) modifies miRNAs in melanocytes [261]. During the progression of melanoma, ADAR expression is downregulated. This causes a reduction of adenosine to inosine modifications in miRNAs, which changes the miRNA binding profile to promote tumor growth [261,262]. One of the most abundant post-transcriptional RNA modifications is N6-Methyladenosine (m^6^A)-methylation, which can also affect the levels of different miRNAs [263,264]. The methyltransferases methyltransferase-like 3 (METTL3) and methyltransferase-like 14 (METTL14), which are major responsible proteins for m^6^A-RNA-methylation, were found to be upregulated in HCC in several studies leading to increased tumor growth both in vitro and in vivo [264]. 

Together, current literature provides compelling evidence that genetic and post-transcriptional modifications of miRNAs play important roles for miRNA function in melanoma and HCC cells and are promising targets for tumor therapy (Figure 9).

As for a huge number of tumor-promoting genes, also for miRNAs, the regulation by dysregulated transcription factors plays an important role for aberrant miRNA expression (Figure 9). A prominent example of a miRNA regulated by specific transcription factors is *miR-210*, which is regulated by binding of HIF1α by a specific response element in the miRNA-precursor sequence in melanoma [221,231] and also in HCC [215]. Furthermore, when melanoma cells become metastatic, the transcription factor ETS-1 gets phosphorylated and promotes transcription of *miR-222* [265].

Another example comprises the homeodomain-containing transcription factors HOXB7/PBX2, which are active during embryonic development and are normally silenced in adult cells. However, they get re-activated during melanoma development and *miR-221* is regulated by aberrant expression of these transcription factors [196,266].

In addition, the activation of NF-κB, for example, via the Staphylococcal nuclease domain-containing 1 (SND1), which is upregulated in HCC, induces expression of *miR-221* and leads to subsequent activation of the pro-angiogenic factors angiogenin and CXCL16 [267].

A further important tumorigenic transcription factor is Myc, upregulating numerous oncogenic miRNAs as well as inhibiting tumor-suppressive miRNAs [255,268]. Among others, myc is transcriptionally regulating the *miR-17* family, which is commonly overexpressed amongst many tumor types including HCC [269].

Another example is the transcription factor CCAAT/enhancer-binding protein alpha (CEBPα), a tumor-suppressor protein which plays an important role for normal hepatocyte function. It was targeted by MTL-CEBPA, the first drug based on a so-called “small activating RNA”, a short miRNA-like oligonucleotide promoting transcription from target loci, tested in the clinic [270]. Thus, targeting transcription factors for cancer treatment can strongly influence miRNA expression and function in cancer, thereby representing a further potential futures therapeutic strategy.

### 7.2. Protein Regulators of microRNA Expression and Function

Due to the complex and strongly controlled cascade of miRNA processing and maturation, it is obvious that not only alterations in miRNAs expression themselves but also misexpression of the proteins in the miRNA processing pathway can contribute to cancer development and progression. Obernosterer et al. were the first group that revealed in 2006 that a tissue-specific Dicer activity is regulating mature miRNA levels [283]. The relevance of this mechanism for the melanocytic lineage was shown by the group of Fisher et al. describing a transcriptional regulation of Dicer by MITF during melanocyte differentiation resulting in classes of miRNAs either accumulating as pre-miRNAs or as mature miRNAs [284]. For melanoma, controversial studies exist regarding Dicer expression levels and its correlation to survival [285,286,287,288,289], indicating a specification into different melanoma subtypes regarding Dicer function for melanoma progression.

In HCC, Dicer is significantly downregulated in cancerogenic tissues as compared with non-tumorous liver tissues [290]. This could be a result of hypoxia, which induces downregulation of Dicer both in vitro and in vivo in HCC [274]. Dicer expression in HCC cells is also inhibited by *miR-18a* promoting cell migration and invasion [291]. The tumor-suppressive cytokine melanoma differentiation-associated gene-7/interleukin-24 (*MDA-7*/*IL-24*) inhibits tumor growth, angiogenesis, metastasis and invasion of different types of cancers and has been promisingly tested in a Phase I/II clinical trial [292]. Mda-7/*IL-24* regulates a specific subset of miRNAs, for example, oncogenic *miR-221*, via down-regulation of Dicer [293,294]. Thus, targeting Dicer could be a potentially promising approach for specific tumor types and conditions such as hypoxia (Figure 9).

Drosha processing of specific miRNAs is activated during embryonic development [295]. As a consequence, Drosha processing is blocked in tumorigenesis leading to the reduced expression of a majority of miRNAs in numerous types of cancer. In melanoma, nuclear expression of Drosha protein and mRNA is markedly reduced in the early stages while cytoplasmic expression is increased [271]. This could indicate Drosha as a target against induction of miRNAs driving different stages of tumor-progression (Figure 9).

We identified *XPO5* as significantly overexpressed in melanoma compared to normal human epidermal melanocytes (NHEM), contributing to enhanced survival, proliferation and metastasis of melanoma cells [272]. The enhanced *XPO5* expression is partly due to constitutively active MEK/ERK signaling in melanoma and partly due to increased mRNA stability because of a single nucleotide polymorphism (SNP; rs11077) in the *miR-617* binding site [272]. In HCC, the A/A genotype of the same SNP is associated with worse survival of HCC patients [296]. As siRNA mediated knockdown of *XPO5* leads to reduced levels of plenty of the cellular miRNAs in melanoma [272], it is reasonable to assume that elevated *XPO5* protein levels as seen in melanoma are responsible for the general elevation of miRNA levels which is a quite exclusive feature in melanoma (see section “the role of miRNAs in melanoma and hepatocellular carcinoma”).

We could show that AGO proteins are downregulated in melanoma cells as compared to other cancer-derived cell lines [276]. Thereby, we observed the strongest reduction for *AGO2* [275,276] which normally appeared to be the most abundant AGO protein in human cells [276,297,298]. Furthermore, a strong reduction of siRNA effectivity against different oncogenes in melanoma cells was observed, which aggravates a siRNA or miRNA based therapy in melanoma [275]. In HCC, the E3 ubiquitin ligase Lin-41 is frequently overexpressed, leading to downregulation of its targets *AGO1* and *AGO2*. This affects miRNA abundance and functionality in HCC cells and promotes proliferation [299]. Another study showed that *AGO2* mRNA and protein levels were upregulated in HCC tissues and that *AGO2* expression can be regulated by the tumor-suppressive *miR-99a* [300]. Grimm et al. proved *AGO2* to be the rate-limiting factor for RNAi mechanisms as therapeutic application [301]. They could show in vivo that pre-application of *AGO2* extended the efficiency and persistence of RNAi based agents and also reduced hepatotoxicity [301]. Therefore, improving *AGO2* expression and function might represent a promising approach to support miRNA-based therapeutics by increasing miR-efficiency.

Further proteins majorly influencing miRNA expression and function are the homologs LIN28A and LIN28B. In stem cells, these RNA-binding proteins inhibit the expression of the *let-7* miRNA family via binding to the *let-7* pre-miRNA hairpin thereby prohibiting maturation of this miRNA [302]. In different cancer types, LIN28 can downregulate *let-7* in the same way to prevent expression of this tumor-suppressive miRNA. In melanoma patients, LIN28B is often aberrantly expressed, reveals several oncogenic properties and is functionally required for melanoma progression [303]. Overexpression of Lin28B reduced mature *let-7* miRNA expression resulting in an enhanced sphere-forming ability of melanoma cells (sphere formation is a characteristic stem cell-like in vitro feature of many highly malignant cancer cells) [304]. The reduction of the tumor-suppressive *miR-26a* induces an upregulation of LIN28B, which is a direct target of this miRNA, in diverse cancers including melanoma and HCC. This is accompanied by a *let-7* miRNA downregulation that enhances tumor growth and metastasis [305]. RNAi mediated knockdown of LIN28B decreased proliferation of HCC cells and reduced tumor growth in vivo [306]. Overexpression of LIN28B also induced enhanced tumorigenicity and induction of EMT [306]. In Hepatitis B virus-infected cells, the hepatitis B virus X protein (HBx) mediates overexpression of Lin28B leading to suppression of *let-7* and herewith preparing malignant transformation of hepatocytes [307]. High expression of LIN28 in HCC is further associated with resistance to the chemotherapeutic paclitaxel [308], indicating the importance of the LIN28/let-7 axis for HCC treatment. To inhibit binding of the negative regulator LIN28 to the tumor-suppressive miRNA *let-7*, short, loop-targeting oligoribonucleotides can be used. These so called “looptomiRs” lead to suppression of cancer cell growth and provide a promising therapeutic strategy [273] (Figure 9).

In summary, protein regulators of miRNA processing and function strongly impact expression and efficiency of miRNAs and thereby. represent further potential therapeutic targets in cancer.

## 8. Therapeutic Targeting of miRNAs and miRNA-Pathways

Since miRNAs are small RNA oligonucleotides, the most obvious way to inhibit for example, their oncogenic effect is the use of complementary RNA molecules binding to the respective miRNA thereby inhibiting its mRNA-binding function. In contrast to oncogenic miRNAs, single tumor-suppressive miRNAs that are lost during tumor development can be replaced using miRNA mimics. The problem with such miRNA mimics or anti-miRs, respectively, which consist of naturally occurring RNA components, is that they have an only low binding affinity and show poor resistance against intracellular nucleases and degradation [309]. For therapeutic use, it is better to use chemically modified RNA molecules, for example, locked nucleic acids (LNAs) [281] (Figure 9). LNAs comprise an extremely high affinity to their targets, a high sensitivity regarding mismatches and a good stability [309]. A LNA was used as the first miRNA based drug entering a clinical study—Miravirsen is a complementary molecule targeting *miR-122* [310]. Miravirsen was well tolerated with no dose-limiting toxicities in a Phase I clinical study; in a follow up Phase II study treatment with Miravirsen provided dose-dependent and long-lasting antiviral activity in treatment-naive patients with chronic HCV infection [310].

As comprehensively depicted above, one single miRNA can regulate multiple targets [15,88,91,311]. Systemic inhibition of a defined miRNA in melanoma or HCC patients could therefore also lead to adverse side effects. Because of this, a considerable alternative approach would be to specifically interfere with single miRNA-target gene interactions by using for example, an LNA masking the specific miRNA binding site on only one specific target gene of interest. In the very same manner, Cibois et al. proved this concept by designing a membrane permeable, modified oligonucleotide that suppresses the binding of CUG-binding protein 1 to the mRNA of Su(H). The latter is a key molecule in the notch signaling pathway and this approach influenced the development of Xenopus laevis embryos [312].

Another possibility to clinically target miRNAs is the use of small-molecule inhibitors (Figure 9). A reporter gene-based screen with over 300,000 different compounds lead to the identification of for example, a specific and efficient inhibitor of *miR-21* transcription inducing apoptosis of the cervical carcinoma cell line HeLa and preventing assembly of microtumors in low doses in vitro [280].

A further promising way to therapeutically influence miRNA pathways is to re-express specific miRNAs, for example, using virus-based systems. The systemic delivery of adeno-associated viruses carrying *miR-26a* into mice with HCC tumors caused a strong reduction of cancer cell proliferation and increased apoptosis of tumor cells leading to diminished disease progression without toxicity to healthy tissues [313].

Another study used adeno-associated viral vectors (AAVs) carrying multiple binding sites for *miR-221* to sequester endogenous *miR-221* cellular molecules [314]. This led to an increase in CDKN1B/p27 protein expression and enhanced apoptosis of HCC cells [314].

## 9. Delivery Strategies of miRNA-Associated Therapeutics

Treatment of patients with siRNAs or miRNAs for therapeutic purposes leads to certain risks. Free RNA molecules will be easily degraded by cellular nucleases and can negatively influence the immune system. Furthermore, caused by their negative charge, siRNAs or miRNAs can hardly pass the cell membrane [282]. Therefore, a lot of research effort was made in recent years to optimize delivery strategies for RNAi bases therapeutics. One promising transfer method for RNA molecules is a nanoparticle-based system (Figure 9). The RNA in a nanoparticle is protected from external influences and the particles can be chemically modified to improve target cell specificity [282]. Nanoparticles are between 1–100 nm in diameter. They can be built using positively charged lipids surrounding the RNA as well as positive-charged polymers or silica, which can be equipped with small pores, where drugs assisting delivery and RNase protection can be attached [281]. The first siRNA-based drug successfully tested in a Phase I clinical study against solid tumors (the study was investigating melanoma patients) using a nanoparticle-based delivery system was CALAA-01 [261]. The siRNA targets the M2 subunit of the ribonucleotide reductase which plays an important role during DNA replication and is therefore essential for fast replicating cancer cells. CALAA-01 is coated with molecules recognizing the transferrin receptor which is strongly expressed on the surface of cancer cells, ensuring targeted uptake of the drug [315]. Indeed, in this study, systemic delivery of siRNA via targeted nanoparticles was proven to be safe and induced specific, siRNA-mediated gene silencing. However, no objective tumor responses were detected [315].

A further example for a nanoparticle-based siRNA drug is ALN-VSP, consisting of two siRNAs targeting the vascular endothelial growth factor (VEGF) and the kinesin spindle protein (KSP) and being successfully tested in Phase I for treatment of advanced solid liver associated tumors [316]. Among 24 evaluable patients, 4 reached a state of stable disease or even improvement after treatment with ALN-VSP [316].

A hyaluronic acid-modified, polyetherimide-conjugated PEGylated gold nanocage ternary nanocomplex carrying the *miR-26a* could accumulate in the liver in an orthotopic mouse model of HCC for a longer time than in normal mice and could significantly reduce tumor growth under near-infrared radiation [317]. A negatively charged liposomal delivery system with a mean particle size of 122.5 nm was used for intravenous injection in an HCC xenograft mouse model to deliver anti-*miR-221* oligonucleotides and could efficiently increase the expressions of the *miR-221* targets *PTEN*, *P27(kip1)* and *TIMP* [318].

Besides nanoparticle-based delivery strategies, also other modifications of siRNAs or miRNAs to improve cellular uptake have been tested (Figure 9). A cholesterol-conjugated *let-7a* miRNA mimic showed a high transfection efficiency in human HCC cells and a high affinity for liver tissue in vivo after systemic treatment of mice [319]. A cholesterol-modified isoform of anti-*miR-221* showed improved pharmacokinetics and delivery to liver tissue in mice compared with the unmodified version. It significantly reduced *miR-221* levels and tumor cell proliferation, increased apoptosis of tumor cells and prolonged survival of the mice [320].

SiRNAs or miRNA mimics can also be directly delivered to cells when they are conjugated to N-acetyl-D-galactosamine (GalNAc) and are taken up via clathrin-mediated endocytosis [33]. GalNAc-miRNAs are preferentially taken up by liver cells due to a high affinity for the asialoglycoprotein receptor and are thus particularly suitable for therapy of liver diseases [33].

Moreover, it was widely demonstrated that the cargo of Extracellular Vesicles (EVs), of which exosomes are the most studied, are enriched with miRNAs which play crucial roles in cancer diagnostics, prognosis and also therapeutic approaches [321,322]. Although clinical application of EV-associated miRNAs is still in its infancy, several studies have demonstrated their potential role in preclinical cancer models [321]. For example, the exomiRNA cytotoxic signal delivered from NK to tumor cells was shown to reduce tumor growth [321]. Recently, Neviani et al. showed that NK-mediated killing of neuroblastoma cells is, at least partly, mediated by the transfer of *miR-186* in EVs [321,323]. Moreover, in this study, in vivo activity of *miR-186*-loaded anionic lipopolyplex nanoparticles directed against neuroblastoma cells through their coating with anti-GD2, a neuroblastoma marker, was proven to be sufficient [321]. Furthermore, the first clinical trials were performed evaluating the potential of miRNA delivery by EVs. The first phase I trial of a liposomal *miR-34a* mimic, namely MRX34, was performed in HCC patients and has been published in 2017 [321,324]. Furthermore, miRNA-loaded minicells—called TargomiRs—were used in patients (phase I trial) with recurrent malignant pleural mesothelioma [325]. Here, TargomiRs were loaded with miR16-based mimic miRNA, targeting Epidermal Growth Factor (EGFR). However, the trial reported five dose-limiting toxicities including cardiac ischemia, cardiomyopathy, infusion-related reaction, non-cardiac pain and anaphylactoid reaction, as well as adverse events like transient lymphopenia and increased transaminases [321,325]. Together, a rising number of preclinical models as well as first clinical trials investigate the potential therapeutic application of the concept of EV-containing miRNAs. However, at this timepoint, it is too early to draw conclusions, especially regarding safety and efficacy as well as potential drawbacks of this exciting technology in cancer therapy.

## 10. Cooperative Action with Existing Therapies

Numerous studies show an improved function of classical chemotherapy, targeted therapy or immunotherapy in combination with miRNA function. MiRNA inhibitors or mimics could thus be used in combination with other therapeutic agents to improve therapy outcomes.

Serguienko et al. could show that the enhanced metabolism caused by *let-7* transfection in melanoma cells leads to a higher sensitivity of the cells to the anti-cancer drug doxorubicin, which can induce ROS-production and apoptosis [94]. A recent study confirmed that overexpression of **let-7b** and **let-7c** increased the sensitivity to chemotherapeutic treatment in melanoma [130]. *MiR-204* and *miR-211* play a role for targeted therapy of melanoma as they can contribute to the resistance of melanoma cells to treatment with the BRAF inhibitor Vemurafenib [326]. Furthermore, a successful Phase I study applied siRNAs against the immunoproteasome, which modifies antigen processing by the proteasome in dendritic cells, thus improving recognition of tumor cells and enhancing the T-cell response against the cancer cells [327]. Moreover, the design of pharmacologic inhibitors to directly or indirectly tackle these target genes was proven to be successful in many studies and also showed cooperative effects. For example, we have revealed wildtype KRAS as potent *miR-622*-target gene. KRAS mediated the effects of a loss of this miRNA both in HCC and in melanoma and we demonstrated strong anti-tumor effects of the novel small molecule inhibitor of KRAS, deltarasin, in HCC and melanoma in vitro and in vivo [5,109,118]. Moreover, combinatory approaches of KRAS inhibition (applying *miR-622* or RNAi-mediated or pharmacologic KRAS-inhibition) and sorafenib in HCC or vemurafenib in melanoma, respectively, revealed synergistic anti-tumorigenic effects and reverted chemoresistance in both cancer types [5,109,118]. This highlights a common and conserved function of *miR-622* in cancer biology.

Besides classical chemotherapy, there are also hints that miRNA agents can function in combination with innovative therapeutic approaches. Myrothecine A, a substance extracted from a fungus found in the traditional Chinese medicinal plant *Artemisia annua*, was revealed to inhibit the *miR-221*-induced cell proliferation of HCC cells and to release p27 protein expression by inhibiting *miR-221* [328]. Another naturally occurring compound, α-pinene, induced cell cycle arrest via inhibition of *miR-221* expression and promoted antitumor activity in HCC cells [329]. Furthermore, the traditional Chinese medicines astragaloside IV and curcumin lowered the levels of *miR-221* in HCC and significantly reduced mean tumor weight in an orthotopic nude-mouse model of human HCC [330].

Together, numerous therapeutic strategies including modified or non-modified miRNA-mimics, miRNA inhibitors and innovative delivery strategies, pharmacologic or RNAi-mediated target-gene inhibition strategies, masking of specific miRNA-binding sites or combinatory approaches applying these miRNA-based therapies together with chemo- and immunotherapy mark most promising novel options for cancer patients in the future.

## 11. Conclusions and Future Challenges

The described mechanisms and approaches for using miRNAs as therapeutic tools open up fascinating and highly promising options for future cancer therapies. Still, there are many unanswered questions to realize the full therapeutic potential of miRNAs and miRNA-associated regulators.

Most of the existing clinical studies contain siRNA based approaches to downregulate disease associated genes [16]. Therapeutic application of miRNAs is still in its infancy. Almost all of the most promising miRNA candidates for therapeutic options are still in the preclinical stage [16].

The development of nano-particle based methods made a huge advantage for delivery of miRNA- or siRNA-based molecules. In the described clinical study of CALAA-01, the first siRNA-based drug successfully tested in a Phase I clinical trial, delivery to melanoma cells worked specific and without severe side effects [331]. However, there are open questions regarding stability of the respective particles, endosomal escape for miRNA delivery, biodegradability after miRNA release or the risk of accumulation in the human body [281].

Moreover, another critical concern is that induction of miRNAs via non-viral and viral vectors leads to liver toxicity and death in mice due to oversaturation of cellular miRNA pathways [332]. This can even induce HCC [333].

Furthermore, the high complexity of the miRNA pathways is a major obstacle for specific miRNA-associated therapeutic approaches. As described in this review, one miRNA regulates many target genes of different pathways. This makes inhibition of miRNA function as therapeutic tool not completely foreseeable and bears risks of adverse side effects. Therefore, as stated above, therapeutic miRNAs should majorly act solely as tumor-suppressors or oncogenes in one specific setting to avoid mutual neutralization effects. The miRNAs which were presented in this review were proven to be “specific” tumor-suppressors or oncogenes, respectively, in two exemplary types of typical miRNA-regulated cancers, melanoma and HCC, as well as in other cancer types, thereby outlining these miRNAs as potential therapeutic tools.

Moreover, numerous clinical studies addressing miRNAs or using siRNAs show promising results regarding delivery and safety but only display poor results concerning tumor treatment [331]. This could be due to the highly interconnected impact of one miRNA to different cellular pathways leading to potentially opposing effects.

In summary, this review presents that melanoma and HCC show similar miRNA related patterns. Important tumor-suppressor or oncogenic miRNAs, which often play pivotal roles during embryonic development, as for example, the *let-7* miRNA family, can be found deregulated during development of these two cancer types as well as in many further types of tumors. Dysregulation of similar miRNAs in different cancer types, descending from completely different origins and risk factors such as melanoma and HCC, demonstrates the importance of miRNA function for tumorigenesis and cancer progression. Novel delivery strategies using targeted delivery mechanisms such as the described nanoparticles or specifically modified oligonucleotides can ensure a precise administration with minimized side effects in the future. Still, as outlined above, many unsolved questions and challenges regarding therapeutic approaches should be addressed in futures studies to precisely understand miRNA function, potential delivery strategies and side effects as well as functional connections between single miRNAs and their targets.

Taken together, the world of those small, regulatory molecules constitutes one of the most exciting, innovative and dynamic fields in cancer research and might markedly improve futures cancer therapies.

## Figures and Tables

**Table 1:** References Depicting Differential Expression of single *Let-7* Family Members in Melanoma and HCC.

*Let-7* Family Member	References Showing Downregulation in Melanoma	References Showing Upregulation in Melanoma	References Showing Downregulation in HCC	References Showing Upregulation in HCC
*Let-7a*	[4,88,92,93,126,127]	-	[41,106,128,129]	-
*Let-7b*	[4,12,93,130]	[127]	[41,106,128,129]	-
*Let-7c*	[88,126,130]	[127]	[41,106,128,129,131]	-
*Let-7d*	[36,93,127]	-	[41,106,129]	-
*Let-7e*	[88,93]	[126]	[106,129]	-
*Let-7f*	[36,126,127]	-	[106,129]	-
*Let-7g*	[93,126,127]	-	[106,129,132,133]	-
*Let-7i*	[36,127]	[126]	[129,132]	-
